# Early inflammatory response in epithelial ovarian tumor cyst fluids

**DOI:** 10.1002/cam4.282

**Published:** 2014-06-20

**Authors:** Björg Kristjánsdóttir, Karolina Partheen, Eric T Fung, Christine Yip, Kristina Levan, Karin Sundfeldt

**Affiliations:** 1 Department of Obstetrics and Gynecology, Institute of Clinical Sciences, University of GothenburgGothenburg, Sweden; 2 Vermillion, Inc.Fremont, California

**Keywords:** Biomarkers, cytokines, epithelial ovarian cancer, ovarian cyst fluid, proteomic

## Abstract

Mortality rates for epithelial ovarian cancer (EOC) are high, mainly due to late-stage diagnosis. The identification of biomarkers for this cancer could contribute to earlier diagnosis and increased survival rates. Given that chronic inflammation plays a central role in cancer initiation and progression, we selected and tested 15 cancer-related cytokines and growth factors in 38 ovarian cyst fluid samples. We used ovarian cyst fluid since it is found in proximity to the pathology and mined it for inflammatory biomarkers suitable for early detection of EOC. Immunoprecipitation and high-throughput sample fractionation were obtained by using tandem antibody libraries bead and mass spectrometry. Two proteins, monocyte chemoattractant protein-1 (MCP-1/CCL2) and interleucin-8 (IL-8/CXCL8), were significantly (*P* < 0.0001) higher in the malignant (*n* = 16) versus benign (*n* = 22) tumor cysts. Validation of MCP-1, IL-8, and growth-regulated protein-*α* (GRO*α*/CXCL1) was performed with ELISA in benign, borderline, and malignant cyst fluids (*n* = 256) and corresponding serum (*n* = 256). CA125 was measured in serum from all patients and used in the algorithms performed. MCP-1, IL-8, and GRO*α* are proinflammatory cytokines and promoters of tumor growth. From 5- to 100-fold higher concentrations of MCP-1, IL-8 and GRO*α* were detected in the cyst fluids compared to the serum. Significant (*P* < 0.001) cytokine response was already established in borderline cyst fluids and stage I EOC. In serum a significant (*P* < 0.01) increase of IL-8 and GRO*α* was found, but not until stage I and stage III EOC, respectively. These findings confirm that early events in tumorigenesis can be analyzed and detected in the tumor environment and we conclude that ovarian cyst fluid is a promising source in the search for new biomarkers for early ovarian tumors.

## Introduction

EOC represents a very heterogeneous disease with widely varying histology, pathogenesis, and clinical behavior 1. The majority of epithelial ovarian cancers (EOC) contain cystic and solid components, and less than 15% are without cysts 2. The ovarian cyst fluid in the center of tumor activity most likely represents the local microenvironment of the ovarian tumor 3. The malignant ovarian cyst fluid contains high concentrations of proteins 4–6. It is thus possible that biomarkers in the ovarian fluid might reflect events in ovarian tumorigenesis earlier than biomarkers in the peripheral blood circulation 3. The role of inflammation in carcinogenesis was suggested by Virchow in the 19th century 7, and there is now growing evidence that an inflammatory microenvironment is an essential component of tumor progression 8,9. Cancer-related inflammation (CRI) is postulated to be one of the seven hallmarks of cancer 10,11. Complex cytokine–chemokine networks regulate and modulate inflammation in EOC, where chemokines are mediators of a number of biological processes, including inflammation, angiogenesis, and cell migration 12.

Development of epithelial ovarian neoplasm has been related to the process of ovulation and increased ovulations per life increase the risk of getting EOC 13. Ovulation is considered to be an inflammatory process which involves repeated minor trauma to the ovarian surface and exposure to estrogen-rich follicular fluid, cytokine release, influx of inflammatory cells to the ovarian stroma, production of reactive oxygen and nitrogen species (ROS and RNS), and remodeling of the extracellular matrix to repair the wound 13. In this process, epithelial cells undergo epithelial-to-mesenchymal transition (EMT) with altered proliferative response and enhance inflammation. These transformed cells are more like fibroblasts with increased motility and invasion ability 8,14. Immunity shows dual activity, called “cancer immunoediting,” being both host protective and tumor promoting 15. Invaginations of the ovarian surface epithelium and accumulation of inclusion cysts are associated with more ovulations and establish an environment that favors tumor cell growth 16. Ovarian surface implants from fallopian tube carcinomas have been proposed to form part of the initiation of ovarian carcinoma 17. These implants from the tubal fimbria may remain clinically occult within the inclusion cysts and invaginations before detection 18,19.

EOC comprises the majority of malignant ovarian tumors and is one of the most deadly cancers among women in the Western world. Each year approximately nine of 100,000 women will be diagnosed and five of 100,000 will die from EOC 20. Age, family history, previous breast cancer, mutations in BRCA1 or BRCA2 genes, low parity, and more ovulations over a lifetime further enhance the risk 21. Unspecific early symptoms, the localization of the ovaries deep in the pelvis which causes difficulties in palpation, and the lack of sensitive early markers contribute to late diagnosis 22. According to the International Federation of Gynecology and Obstetrics (FIGO), the 5-year survival rate for stage III is only 25–30%, whereas for patients diagnosed early when the cancer is still confined to the ovaries (stage I), the 5-year survival rate is more than 90% 23,24. Therefore, early detection of EOC has great promise to improve clinical outcome.

Numerous studies have been undertaken to find an alternative to the current 30-year-old tumor marker, cancer antigen 125 (CA125) 25. CA125 is positive in about 80% of malignant tumors, but only in 50–60% of early-stage EOC. It is elevated in various benign gynecologic conditions like endometriosis and also in nongynecologic conditions as well in premenopausal women 26–28. We urgently need more specific and selective biomarkers to improve early diagnosis of EOC. Proteins that are involved in the inflammatory response may serve as early EOC biomarkers, even though they are not tumor specific.

We studied ovarian cyst fluid from benign, borderline, and malignant cystic epithelial ovarian tumors in order to identify inflammatory biomarkers for early diagnosis of EOC. Exploration of the immunoproteome of ovarian cyst fluid from 38 patients was performed by a direct targeting method: immunoprecipitation with selected monoclonal antibodies, specific for 15 cytokines and growth factors known to be involved in the immune response in cancer. This was followed by mass spectrometry (MS). Identified proteins with the highest significance, Receiver operator characteristic (ROC), area under the curve (AUC), and the greatest increase in fold change were chosen for validation in a larger set of ovarian cyst fluid (*n* = 256) and corresponding blood serum (*n* = 256). Data were related to serum levels of the established EOC biomarker CA125. CA125 were measured in the ovarian cyst fluid as well and correlated with the serum levels.

## Materials and Methods

### Clinical samples

Cyst fluids and blood samples were collected prospectively and consecutively from 291 women presenting with suspected malignant ovarian cysts from March 2001 to September 2007 (Table 1). All the patients had been admitted to the unit for gynecologic oncology surgery at Sahlgrenska University Hospital, Gothenburg, Sweden. According to our protocol blood samples were taken after anesthesia but prior to surgery. Ovarian cyst fluid was aspirated directly after removal of the cysts from the abdomen. All samples were immediately cooled to 4°C for 15–30 min, centrifuged, aliquoted into Eppendorf tubes, and stored at −80°C within 30–60 min of collection. Samples used in this study had one freeze–thaw cycle. We were unable to analyze 20 ovarian cyst samples (*n* = 13 benign, *n* = 3 borderline, *n* = 3 EOC due to small amount of material. One sample was borderline dermoid with to high viscosity). Fifteen other samples turned out to be metastasis from cancers other than EOC. The remaining samples from 256 women were available for analysis. CA125 was measured in the blood samples from all patients with ovarian cysts. The tumors were staged according to FIGO classification and histopathology, and grade was established by an experienced pathologist (Table 2). The study had been approved by the local ethics committee at the University of Gothenburg and each patient gave their informed written consent. Handling and processing of samples were standardized for all patients.

**Table 1:** Sample characteristics of (A) immunoprecipitation–MS cohort and (B) validation cohort

A	Benign	Borderline	Malignant	Grade		FIGO Stage		
Histology				G1/G2/G3	I	II	III	IV
*n* = 38 (%)	*n* = 22 (58)	*n* = 0	*n* = 16 (42)					
Simple	5							
Stromal	2							
Dermoid	2							
Endometrioma	2							
Serous	7		11	1, 5, 5	4	1	6	
Endometrioid			2	2, 0, 0	2			
Clear cell			1	1, 0, 0			1	
Undiff			2	1, 0, 0	1		1	

B	Benign	Borderline	Malignant	Grade		FIGO Stage		
Mean age (range)	60 (16–88)	51 (31–85)	61 (28–88)					
Histology				G1/G2/G3	I	II	III	IV
*n* = 256 (%)	*n* = 156 (61)	*n* = 22 (9)	*n* = 78 (30)	27/17/27	*n* = 32 (41)	*n* = 7 (9)	*n* = 36 (46)	*n* = 4 (4)
Simple	42 (27)							
Stromal	11 (7)							
Endometrioma	6 (4)							
Hemorrhagic	1 (1)							
Dermoid	8 (5)		1 (1)	0, 0, 1		1		
Serous	54 (35)	12 (54)	48 (61)	13, 13, 22	15	4	27	2
Mucinous	34 (22)	9 (41)	7 (9)	6, 1, 0	4	1	1	1
Endometrioid		1 (5)	9 (12)	4, 2, 3	5	1	3	
Clear cell			6 (8)	4, 1, 1	4		2	
Undiff			4 (5)		1		3	
Granulosa cell			3 (4)		3			

G1, highly; G2, moderately; G3, poorly differentiated.

**Table 2:** Tumor marker levels in cyst fluids and serum

	Benign (B) *n* = 156 median (range)	Borderline (BOT) *n* = 22 median (range)	B versus BOT *P*-value	M stage I (Ml) *n* = 32 median (range)	B versus Ml *P*-value	All malignant (M) *n* = 78	B versus M *P*-value
*Cyst fluid*							
GROa	343 (11–1435)	921 (140–1403)	<0.001*	922 (15–1430)	0.003*	919 (15–1430)	<0.001*
IL-8	756 (1–279,000)	16,712 (99–757,000)	<0.001*	11,903 (11–530,000)	<0.001*	6704 (11–530,000)	<0.001*
MCP-1	10,512 (38–184,000)	93,670 (29–390,000)	<0.001*	27,824 (309–424,000)	0.003*	20,464 (309–424,000)	0.006*
*Serum*							
GROa	58 (18–923)	73 (18–81)	0.09	59 (30–145)	0.411	96 (30–392)	<0.001*
IL-8	7 (3–278)	8 (2–48)	0.590	10 (3–82)	0.006*	13 (3–82)	<0.001*
MCP-1	287 (67–7430)	261 (103–820)	0.75	257 (100–891)	0.303	267 (71–1251)	0.99
CA125	18 (3–716)	52 (7–323)	<0.001*	52 (8–807)	<0.001*	153 (8–955)	<0.001*

GROa, IL-8, and MCP-1 (pg mL); CA125 (U/mL).

* *P* < 0.05.

### Patient material

For the primary analysis, ovarian cyst fluid from 38 women (*n* = 22 benign and *n* = 16 EOC) was used to mine the inflammatory profile of the ovarian cyst fluid (Table 1). The samples were chosen based on heparin profiles, volumes, and clarity/viscosity. The validation cohort consisted of ovarian cyst fluid and corresponding serum from 256 patients, including the 38 patients from the primary analysis (*n* = 156 benign, *n* = 22 borderline, *n* = 74 EOC, 1 malign dermoid, and 3 granulose cells cancers) (Table 1).

### Immunoprecipitation–mass spectrometry

To strengthen our material and to focus on the inflammatory profile of ovarian cysts, we selected 15 cytokines and growth factors known to be involved in the immune response in cancer and used a direct targeting method, immunoprecipitation, to enrich the desired inflammatory proteins in the ovarian cyst fluid. We performed high-throughput sample fractionation by tandem antibody libraries bead, in combination with surface-enhanced laser desorption/ionization time of flight (SELDI). In this approach, potential biomarkers in ovarian cyst fluid are targeted by selected monoclonal antibody mixtures.

We used 38 cyst fluid samples and two antibody mixtures (AB mix I and AB mix II). The division of the antibodies into two groups was based on molecular weights and some structural motifs of the antibodies (Tables 1 and S1).

From each sample, 400-*μ*L cyst fluid was added to 300 *μ*L 150 mmol/L TrisHCl, 0.15 mol/L NaCl, and 0.1% Tween 20 containing protease inhibitor cocktail (Roche, Branford, CT) with 50 *μ*L 50% v/v immobilized Antibody Mix I agarose beads (anti-human IL-8, anti-human IL-1*β*, anti-human regulated on activation normal T cell expressed and secreted, anti-human MCP-1, anti-human MIP1*α*, anti-human GRO*α*, anti-human SDF1) in an perfluorodecyltrichlorosilane (FDTS)-modified fritted 96-deepwell filter plate (Nunc, Thermo Fisher Scientific Inc., Waltham, MA). The plate was sealed, shaken at 4°C for 8 h, and then centrifuged at 1500 rpm for 2 min to collect the unbound fraction. The entire unbound fraction was transferred to 50 *μ*L 50% v/v immobilized Antibody Mix II agarose bead (anti-human IL-6, anti-human IL-12, anti-human transforming growth factor-*β*, anti-human tumor necrosis factor-*α*, anti-human vascular endothelial growth factor, anti-human basic fibroblast growth factor, anti-human granulocyte colony stimulating factor, anti-human granulocyte macrophage colony stimulating factor) in a FDTS-modified fritted 96-deepwell filter plate (Nunc). The plate was sealed, shaken at 4°C for 8 h, and then centrifuged at 1500 rpm for 2 min to collect and remove the unbound fraction. Each well of the 96-well filter plates was washed twice with 500 *μ*L 1 mol/L urea, 0.1% CHAPS (a detergent used to solubilize proteins; 3-[(3-cholamidopropyl) dimethylammonio]-1-propanesulfonate), and phosphate-buffered saline at pH 7.5.

Proteins bound to the antibody Mix I and Mix II beads were differentially eluted, firstly with 100 *μ*L of 50% isopropanol/acetonitrile (2:1), 0.5% formic acid, and 1% trifluoroacetic acid to yield Fraction B1, secondly with 100 *μ*L 3 mol/L guanidine thiocyanate and 0.5% Triton X100 (90°C) to yield Fraction B2a, and thirdly with 100 *μ*L 2 mol/L thiourea, 4 mol/L urea, 1% CHAPS, and 50 mmol/L Tris-HCl pH 7.5 to yield Fraction B2b.

A 30-*μ*L aliquot of each eluted fraction was added to 200 *μ*L of buffer in a bioprocessor containing either a cation exchange ProteinChip array (CM10; Ciphergen, Fremont, CA) or an immobilized Cu ProteinChip array (IMAC30; Ciphergen). After 45 min of incubation with shaking, the arrays were washed once with 200 *μ*L of the buffer and then rinsed with water. After adding sinapinic acid, the chip-bound proteins were profiled using a PCS4000 mass spectrometer (Ciphergen).

Data were acquired using CiphergenExpress (Fremont). Mass calibration was performed using external calibrants. Intensity normalization was based on the total ion current using an external normalization factor, and baseline subtraction was performed. Peak detection was performed in CiphergenExpress using the criteria that a peak must have a signal/noise ratio of 3:1 and be present in 20% of the spectra.

### Validation—ELISA

IL-8 and MCP-1, the two identified proteins with highest significance in the immunoprecipitation–MS analyses, were further validated individually and together with the currently used marker CA125. GRO*α* previously reported to be one of the most highly induced pro-inflammatory cytokines and a potential tumor marker for EOC 28–30 was also included in the validation.

ELISA was performed on ovarian cyst fluid and serum from 256 patients (*n* = 512 samples) (Table 1b). Levels of IL-8, MCP-1, and GRO*α* in pg/mL were determined using the ELISA kits Quantikine® (R&D Systems, Minneapolis, MN) according to the manufacturer's instructions with minor modifications. CA125 in U/mL was measured in blood samples with ELSA-CA125 (Cisbio Bioassays, Codolet, France) according to manufacturer's instruction.

### Statistical analyses

Statistical analyses were performed in SPSS for Windows (version 17), CiphergenExpress (Ciphergen Biosystem) and Prism 5.0 (GraphPad software, La Jolla, CA). The Mann–Whitney *U*-test was used to identify peaks with *P* < 0.001 comparing benign and malignant ovarian cysts in the immunoprecipitation–MS. The protein levels measured with ELISA were subjected to the Mann–Whitney *U*-test and logistic regression analysis. The regression models were estimated for each marker individually and in combinations to differentiate between patients with benign, borderline, and malignant cysts. ROC was constructed for each model, and the AUC was calculated. A level of *P* < 0.05 was considered statistically significant.

## Results

### Two proteins were selected from the enriched immunoprecipitiation–MS

**Figure 1:**
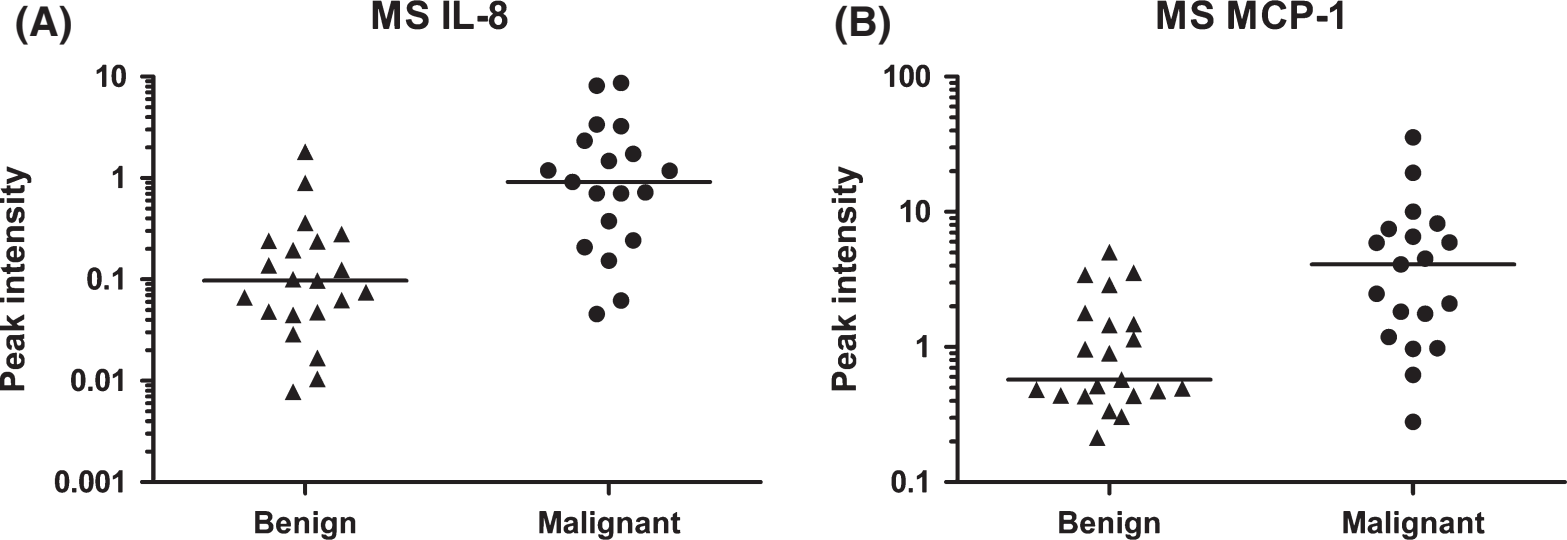
Scatterplots of peak levels (log-scale) for (A) interleukin-8 (IL8) and (B) monocyte chemoattractant protein-1 (MCP-1). Line indicates median.

In the primary experiment performed on (*n* = 38) cyst fluid samples, 150 high-quality peaks were detected as differently expressed between benign and malignant samples (*P* < 0.001). Two of the proteins identified by the immunoprecipitation–MS were MCP-1 and IL-8, with ROC AUC values of 0.82 and 0.80, respectively, and a seven-fold difference in expression. MCP-1 (SwissProt # P13500) had a calculated molecular weight (MW) of 8664.03 Da. IL-8 (SwissProt # P10145) was found in two forms: a full-length protein with a calculated MW of 8918.45 Da, and a truncated form IL-8 (6-77), missing 5 amino acids from the N-terminal, with a calculated MW of 8381.77 Da (Fig.1). Consequently these two potential biomarkers with a higher expression among the malignant samples were selected for further validation with an independent method in both ovarian cyst fluid and serum. The potential biomarker GRO*α* was also included in the validation. Selected biomarkers were related to the performance of CA125 measured in serum of corresponding patients.

### Higher expression of inflammatory proteins was found in ovarian cyst fluid than in serum

**Figure 2:**
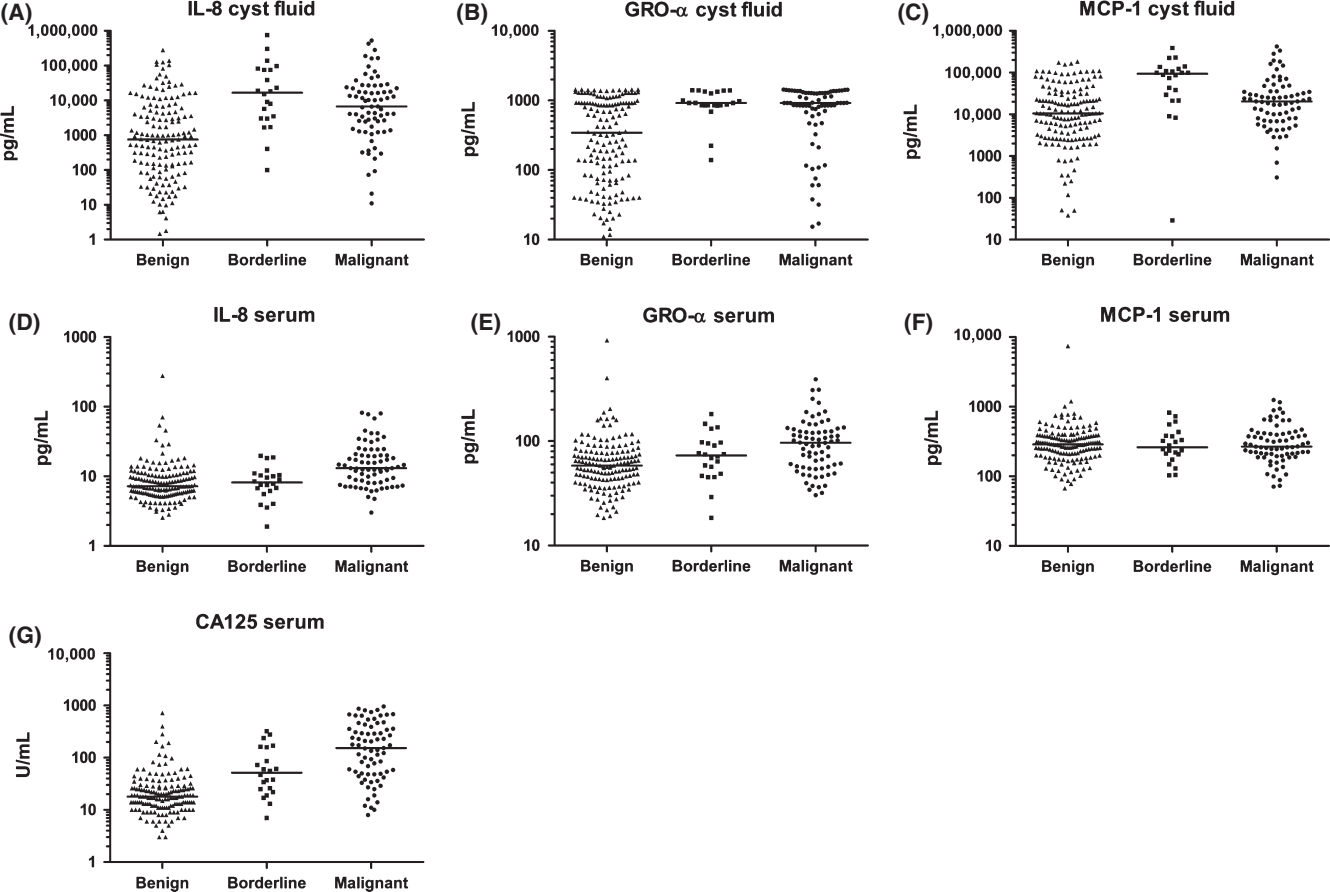
Protein levels measured with ELISA in ovarian benign, borderline, and malignant cystic fluids and serum from corresponding patients (log-scale). Line indicates median. (A–C) Cyst fluid levels of IL-8, GRO*α* and MCP-1, and (D–G) Serum levels of IL-8, GRO*α*, MCP-1, and CA125. MCP, monocyte chemoattractant protein; IL-8, interleukin-8.

The benign ovarian cyst fluid contained higher levels of the cytokines than in serum GRO*α* (median 343 vs. 58), IL-8 (756 vs. 7), and MCP-1 (10512 vs. 287 pg/mL) (Table 2, Fig.2). The same pattern was found in patients with malignant cysts: GRO*α* (median 919 vs. 96), IL-8 (6704 vs. 13), and MCP-1 (20,464 vs. 267 pg/mL). The protein concentrations in serum and cyst fluid from the same patient did not correlate. The age of patients in the benign group was almost as high as in the malignant group with a mean of 60 compared to 61 years. Patients with borderline tumors were 10 years younger, at 51 years (Table 1). Protein levels did not correlate with the age of the patients. The patients in this study are a high-risk population, scheduled for removal of a cystic tumor suspected to be cancerous. Older patients in general are at higher risk of cancer and will therefore be referred to a specialized center. The fact that these patients are a high-risk group could explain the relatively high age in the benign group.

### Higher expression of cytokines in ovarian cyst fluid compared to serum in early tumors

Significantly higher expression of the cytokines was found in the malignant EOC than in benign samples for both cyst fluid and serum samples, with the exception of MCP-1 in serum.

In order to detect variations at an early stage of the disease, we compared benign tumors with borderline tumors and with stage I EOC.

The three chemokines validated in cyst fluid were significantly (*P* < 0.001) differentially expressed comparing benign (*n* = 156) to borderline tumors (*n* = 22). CA125 was the only marker that was significantly (*P* < 0.001) differently expressed in serum from borderline tumors (Table 2). A multiple regression analysis resulted in the best ROC AUC of 0.86, combining our markers in cyst fluid with serum CA125, with MCP-1 as the only independent marker (*P* = 0.004). In serum, the combination of all markers resulted in ROC AUC of 0.78, the same as for CA125 alone (Table 3).

**Table 3:** ROC AUC and sensitivity at fixed specificity 75% for each biomarker and combinations

	B versus BOT ROC AUC (95% CI)	B versus M stage I ROC AUC (95% CI)	B versus all M ROC AUC (95% CI)	Sensitivity
*Cyst fluid*				
GROa	0.74 (0.66–0.83)	0.67 (0.56–0.78)	0.67 (0.60–0.75)	50
IL-8	0.80 (0.71–0.89)	0.73 (0.62–0.84)	0.73 (0.66–0.79)	54
MCP-1	0.81 (0.71–0.92)	0.68 (0.57–0.78)	0.61 (0.54–0.69)	31
CA125 + GROa	0.80 (0.73–0.88)	0.76 (0.65–0.87)	0.86 (0.81–0.92)	83
CA125 + GROa + IL8 + MCP-l	0.86 (0.79–0.94)	0.77 (0.66–0.88)	0.87 (0.82–0.93)	85
*Serum*				
GROa	0.61 (0.49–0.74)	0.45 (0.34–0.56)	0.71 (0.63–0.78)	58
IL-8	0.47 (0.33–0.60)	0.66 (0.55–0.77)	0.76 (0.70–0.83)	66
MCP-1	0.52 (0.38–0.66)	0.56 (0.46–0.66)	0.50 (0.42–0.59)	29
CA125 + GROa + IL8 + MCP-l	0.78 (0.67–0.89)	0.76 (0.65–0.86)	0.88 (0.82–0.93)	77
CA125	0.78 (0.67–0.89)	0.76 (0.65–0.86)	0.88 (0.82–0.93)	86

B, Benign; BOT, Borderline; M, Malignant ovarian tumors; ROC, receiver operator characteristic; AUC, area under the curve; MCP-1, monocyte chemoattractant protein-1.

IL-8, GRO*α*, and MCP-1 showed significantly (*P* < 0.001, *P* = 0.003 and *P* = 0.003, respectively) higher expression in early-stage disease when compared with cyst fluid samples from patients with a benign cyst (*n* = 156) to stage I EOC samples (*n* = 32). IL-8 was the only marker in serum that was significantly differently expressed *(P* = 0.006) beyond CA125 (*P* < 0.001) in stage I EOC serum (Table 2). The same result was achieved including stage II tumors (*n* = 7) as an “early stage” group (*n* = 39). The multiple regression analysis of the cyst fluid markers together with serum CA125 in stage I tumors resulted in ROC AUC of 0.76, with CA125 as an independent marker (*P* = 0.01). CA125 alone reached the same ROC AUC as the combination of all markers in serum (Table 3).

### GRO*α* and IL-8 levels were elevated in all malignant samples

We compared the protein levels in cyst fluid between benign (*n* = 156) and all malignant samples (*n* = 78). Significant differences were noted in ovarian cyst fluid for GRO*α* (*P* < 0.001), IL-8 (*P* < 0.001), and MCP-1 (*P* = 0.006) (Table 2, Fig.2). In serum, CA125, GRO*α*, and IL-8 showed significant (*P* < 0.001) differences, but MCP-1 (*P* = 0.99) could not be identified in serum.

**Figure 3:**
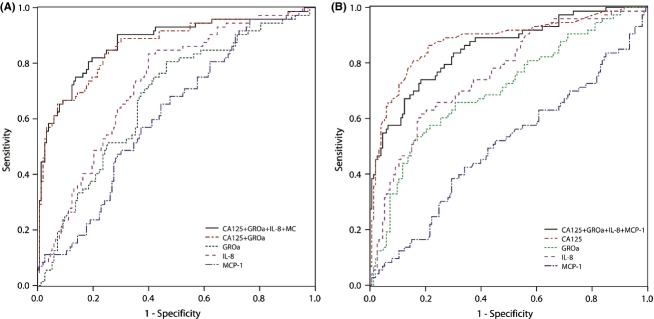
ROC AUC curve for each IL-8, GRO*α*, MCP-1 in (A) ovarian cyst fluid samples, and together with serum CA125 and (B) serum samples. AUC, area under the curve; ROC, receiver operator characteristic; MCP, monocyte chemoattractant protein.

Multiple regression analysis was performed to see whether our markers could contribute to improved diagnosis in patients with ovarian cysts (Table 3). ROC AUC for the three biomarkers in cyst fluid together with serum CA125 was 0.87, with independent markers CA125 (*P* < 0.001), and GRO*α* (*P* = 0.005). In serum our potential biomarkers together with CA125 revealed ROC AUC 0.88, with CA125, IL-8, and GRO*α* as independent markers (*P* < 0.001, *P* = 0.009, *P* = 0.009, respectively). The expression levels of the three biomarkers (GRO*α*, IL-8 and MCP-1) were compared between benign and all malignant tumors. IL-8 was the best chemokine in predicting malignancy, with ROC AUC 0.73 in cyst fluid and AUC 0.76 in serum. However, no other marker combination showed greater ROC AUC values than CA125 as a single marker with ROC AUC 0.88 (Table 3, Fig.3).

We measured CA125 in ovarian cyst fluid and found a correlation between the cyst fluid and the serum samples, with significant higher levels in the ovarian cyst fluid. Pearson correlation factor is 0.477 with *P* = 0.000039 (*P* < 0.001).

## Discussion

Because of its closeness to the primary tumor, ovarian cyst fluid hold promise in the search for novel early ovarian cancer biomarkers. Proteins in the fluid are directly produced, degraded, or secreted by tumor cells, surrounding stroma and immune cells, which are involved in tumorigenesis 3. The aim of this study was to explore proximal fluid and ovarian cyst fluid as a source for early biomarkers and also to identify potential inflammatory biomarkers that could improve the sensitivity of CA125 in early diagnosis of ovarian cancer. Significantly increased inflammatory response reflected by the cytokines studied could already be detected in the cyst fluid of ovarian borderline tumors. However, in serum the increase was not detectable until stage I for IL-8 and stage III EOC for GRO*α*. The cytokines validated were all increased in early-stage EOC in the cyst fluid, but only IL-8 was a significant marker in serum other than CA125.

Chemokines are key players in CRI, and the potential biomarkers analyzed in our study are well characterized as proteins involved in carcinogenesis 29. MCP-1 showed the best performance among all the markers evaluated in the borderline cohort and did better than CA125 (AUC 0.81 vs. 0.78). The upregulation of MCP-1 in cystic fluid early in ovarian tumorigenesis is a very interesting finding of this study. MCP-1 is a potent chemoattractant for monocytes and macrophages, and is involved in a number of inflammatory conditions associated with monocyte recruitment, increased levels of inflammatory cells, and enhanced production of cytokines 30. MCP-1 is a key element of the immunological response to malignant growth, mainly via attraction and activation of tumor-associated macrophages (TAMs). TAMs may promote tumorigenesis by the production of different cytokines 31. It was shown that MCP-1 produced by EOC cells contributes to the local accumulation of TAMs, consequently influencing tumor behavior in both inhibitory and stimulatory manner 32. TAMs are the major players in the connection between inflammation and cancer 33. A previous study by Hefler et al. showed significantly higher MCP-1 serum levels in patients with primary and recurrent ovarian cancer compared to patients with benign ovarian cysts and healthy women 34. We found significantly higher expression in the ovarian cyst fluid in borderline and malignant samples, but our results did not show any difference in MCP-1 in serum. This could be a reflection of the fact that increased levels of MCP-1 in serum have been associated with high-grade and advanced disease 35, but our study consisted of a high percentage (50%) stage I-II EOC.

IL-8 and GRO*α* are both pro-inflammatory chemokines that modulate inflammation and regulate angiogenesis, a process on which tumors depend for growth, survival, invasion, and metastasis 12. This study, like previous studies, shows that IL-8 is elevated in ovarian cyst fluid, ascites, serum, and tumor tissue from ovarian cancer patients, as 4,36. Increased IL-8 and IL-6 expression have been related to poor prognosis 37. IL-8 plays an important role in the development and spread of EOC and influences the migration of different leukocyte populations, endothelial cells, and cancer cells to the tumor site, which explains the high levels of proteins in the ovarian cyst fluid. IL-8 appears to be a link for coagulation and inflammation in malignancy, and it has been associated with a bad prognosis 12. GRO*α* is a potent inducer of senescence in stromal fibroblasts 38. Higher expressions of GRO*α* have been seen in tissue and serum from ovarian cancer patients in comparison to healthy women 39.

Our results show that it may be possible to discriminate between borderline and malignant tumors, even the early malignant tumors from benign ovarian cysts, using MCP-1, IL-8, and GRO*α* as cyst fluid biomarkers.

The high expression of inflammatory proteins in cyst fluid in early-stage EOC supports our theory that inflammatory markers are confined to the actual tumor and to the ovarian cyst in the early process, and only later secrete to the blood circulation 3,5,6. A complex network of cytokines, similar to the ovarian cyst fluid, is present in ascites. The crosstalk between cytokines present in the ascites and the tumor may affect the spread and progression of the disease 40. Higher expression of GRO*α* in ascites than in serum in EOC has been reported, indicating migration of cytokines from the local environment to the peripheral circulation, supporting our hypothesis 41. Since almost 50% of our patients did not present any ascites, we were not able to compare our identified cytokine markers in ascites.

In this study, we focused on inflammatory biomarkers connected to tumorigenesis. We used a direct targeting method, immunoprecipitation with monoclonal antibodies, to avoid or diminish the problem of having high-abundant proteins within the cyst fluid 5, and at the same time enrich selected cytokines and growth factors in our material. Capturing proteins by antibodies serves as a rapid and specific method to select and concentrate specific components 42. It is known that 99% of the plasma proteome are high-abundant proteins and only 1% are low abundant; it is the latter that are the most interesting for early cancer biomarker research 43. The high protein concentration found in the ovarian cyst fluid may indicate that many biomarkers for early detection of ovarian tumors might be present in the cyst fluid. However, until nonrisk needle aspiration techniques are developed 44, aspiration of ovarian cysts is considered hazardous as its risks spreading malignant cells 45.

Specific biomarkers of interest in the cyst fluid could be detected by imaging, for example, with labeled antibodies. This is a speculative but interesting option, not only for early diagnosis but for selection of therapy. Cytokines are multifunctional, with different functionality in different stages of the cancer process. Knowledge about their different functions and pathways is increasing exponentially. Identification of the major mediators and the pathways responsible for triggering an inflammatory response in early stages is not only of interest for diagnosis but also provides opportunities to treat and prevent cancer progression using targeting anti-inflammatory approaches.

Novel ovarian cancer biomarkers are needed to improve the sensitivity and specificity achieved with the currently used biomarker CA125. When distinguishing benign and EOC ovarian cysts, the diagnostic ability of the marker combination in cyst fluid together with serum CA125 (ROC AUC 0.87) was almost the same as for CA125 alone (AUC 0.88). ROC AUC for the marker combination in the early stage EOC was 0.77, compared to 0.76 for CA125 alone.

Early diagnosis of ovarian cancer is very challenging because of the great heterogeneity of the disease, the absence of preclinical lesions in the more aggressive tumors, as well as the relatively low prevalence. Markers that are positive in aggressive tumors are often negative in low-grade tumors and vice versa 46,47. However, the solid and more often highly malignant EOC were not included in this study. The larger cystic tumors are more slowly growing and localized to the ovaries and therefore easier to diagnose early with clinical examination including ultrasound. This can explain to a part the high proportion (*n* = 50%) of early EOC in our cohort. We have to take into account the diversity of the tumor biology of EOC when searching for early cancer markers. A panel with a combination of markers able to cover the multiple variants of EOC could improve early detection. Early markers are likely to be found in proximity to the tumor, as in the case of the low-abundant cytokines in our study.

We conclude that the ovarian cyst fluid, which is in direct contact with the primary tumor, is an excellent source to use in the search for an early EOC biomarker. Five to hundredfold higher concentration of MCP-1, IL-8, and GRO*α* were found in the cyst fluids compared to serum, and the concentration increased with malignancy. Significant cytokine response was already detectable in borderline cyst fluids and stage I EOC. A significant increase in IL-8 and GRO*α* was found in serum, but not until stage I and stage III EOC. In early stage disease, inflammatory markers are still confined to ovarian cystic fluid, but in later stage the cytokines are secreted into the blood. Although the potential biomarkers, MCP-1, IL-8, and GRO*α* are known to be of importance in the carcinogenesis of ovarian cancer, they did not improve on CA125 in early diagnosis.

## Supporting Information

**Table S1.** Antibody mix I and II: selected inflammatory proteins in cancer.
