# Supplementary material for: Early inflammatory response in epithelial ovarian tumor cyst fluids
Source: Cancer Med. 2014 Jun 20;3(5):1302–12. doi: 10.1002/cam4.282 (PMC4302680; doi:10.1002/cam4.282)
Supplement: Supplementary file 1 — Table S1. Antibody mix I and II: selected inflammatory proteins in cancer. [file cam40003-1302-SD1.docx]

**Table S1.** Antibody Mix I and II: Selected inflammatory proteins in cancer
